# Clinical characteristics of Dieulafoy’s lesion in the small bowel diagnosed and treated by double-balloon endoscopy

**DOI:** 10.1186/s12876-023-02913-1

**Published:** 2023-08-24

**Authors:** Masanao Nakamura, Takeshi Yamamura, Keiko Maeda, Tsunaki Sawada, Eri Ishikawa, Kazuhiro Furukawa, Tadashi Iida, Yasuyuki Mizutani, Kentaro Yamao, Takuya Ishikawa, Takashi Honda, Masatoshi Ishigami, Hiroki Kawashima

**Affiliations:** 1 Department of Endoscopy, Nagoya University Hospital, 65 Tsurumai-cho, Showa-ku, Nagoya, 466-8550 Japan; 2 Department of Gastroenterology and Hepatology, Nagoya University Graduate School of Medicine, 65 Tsurumai-cho, Showa-ku, Nagoya, 466-8550 Japan

**Keywords:** Obscure gastrointestinal bleeding, Dieulafoy’s lesion, Balloon-assisted endoscopy, Small bowel, Rebleeding

## Abstract

**Background:**

Obscure gastrointestinal bleeding refers to bleeding for which the source cannot be ascertained even through balloon-assisted endoscopy. In certain instances, Dieulafoy’s lesion in the small bowel is presumed to be the underlying cause.

**Aim:**

This retrospective study aimed to elucidate the clinical characteristics of Dieulafoy’s lesion in the small bowel as diagnosed via double-balloon endoscopy while also exploring the feasibility of predicting bleeding from Dieulafoy’s lesion prior to endoscopy in cases of obscure gastrointestinal bleeding.

**Methods:**

A comprehensive analysis of our database was conducted, identifying 38 patients who received a diagnosis of Dieulafoy’s lesion and subsequently underwent treatment via double-balloon endoscopy. The clinical background, diagnosis, and treatment details of patients with Dieulafoy’s lesion were carefully examined.

**Results:**

The median age of the 38 patients was 72 years, and 50% of the patients were male. A total of 26 (68%) patients exhibited a high comorbidity index. The upper jejunum and lower ileum were the most frequently reported locations for the occurrence of Dieulafoy’s lesion in the small bowel. The detected Dieulafoy’s lesions exhibited active bleeding (n = 33) and an exposed vessel with plaque on the surface (n = 5). Rebleeding after endoscopic treatment occurred in 8 patients (21%, median period: 7 days, range: 1-366 days). We conducted an analysis to determine the definitive nature of the initial double-balloon endoscopy diagnosis. Multivariate analysis revealed that hematochezia of ≥ 2 episodes constituted the independent factor associated with ≥ 2 double-balloon endoscopy diagnoses. Additionally, we explored factors associated with rebleeding following endoscopic treatment. Although the number of hemoclips utilized displayed a likely association, multivariate analysis did not identify any independent factor associated with rebleeding.

**Conclusion:**

If a patient encounters multiple instances of hematochezia, promptly scheduling balloon-assisted endoscopy, equipped with optional instruments without delay is advised, after standard endoscopic evaluation with esophagogastroduodenoscopy and colonoscopy is unrevealing.

## Introduction

Obscure gastrointestinal bleeding (OGIB) is characterized as enigmatic hemorrhaging within the gastrointestinal (GI) tract, even after upper endoscopy and colonoscopy are conducted [1]. Technological advancements in small bowel imaging, including small bowel capsule endoscopy (SBCE), deep enteroscopy, and radiographic imaging, have facilitated the detection of small bowel bleeding in the majority of patients [2–5]. Consequently, the classification of OGIB has been reconsidered, and the utilization of small bowel bleeding as a substitute term has been proposed. The American College of Gastroenterology (ACG) has recommended a clinical guideline that reserves the term OGIB exclusively for patients in whom the source of bleeding remains unidentified throughout the GI tract [6].

Despite the diagnostic progress made, the timely identification of Dieulafoy’s lesion (DL) in the small bowel presents a considerable challenge. DL is characterized by a submucosal artery exhibiting an abnormally enlarged diameter and a tortuous course along the GI lining [7]. This condition was initially described in 1898 by the French surgeon Paul Georges Dieulafoy and has been found to account for 1–2% of all cases of GI hemorrhage [8]. Anatomically, DL is reported to occur most frequently in the stomach (71%), followed by the duodenum (15%), esophagus (8%), rectum (2%), colon (2%), and jejunum (1%) [9, 10]. The aberrant artery typically takes a convoluted path and protrudes (2–5 mm) through the mucosal defect, rendering it susceptible to mechanical damage [11].

Although DL is an exceedingly rare and unusual cause of massive GI bleeding, it can occasionally manifest as a severe and life-threatening condition necessitating prompt intervention. DL in the jejunum can result in recurrent episodes of massive GI bleeding and is not easily diagnosed using conventional methods [12]. This difficulty arises in part from the concealed nature of DLs, which produce intermittent GI bleeding without surrounding mucosal ulceration, making the timely detection of the lesion challenging. In 2004, it was reported that two-thirds of patients with DL were under the age of 40, with the majority presenting with melena [13]. Preoperative identification of the lesion was achieved in 14 out of 41 patients, with angiography proving useful in defining the lesion [13]. Of the 37 patients for whom treatment strategies were reported, 36 underwent surgical treatment. To date, with the advent of balloon-assisted endoscopy (BAE), DL is generally considered an acquired condition, predominantly affecting elderly individuals. Cardoso et al. [14] suggested that DL occurred more frequently in males and in individuals with comorbidities such as cardiopulmonary dysfunction and chronic kidney disease. Several hypotheses have been proposed to explain bleeding from DLs. While the use of aspirin, warfarin, or nonsteroidal anti-inflammatory drugs has been observed in some DL bleeding cases [15], no definitive causal link has been established [14, 16].

Numerous articles have underscored the challenges associated with diagnosing and managing small bowel DLs, and various strategies for approaching refractory bleeding have been suggested [7–9]. Thus far, an Austrian study documenting the treatment of 10 cases of small bowel DL using single or double-balloon endoscopy (DBE) has been published [17]. However, there remain limited data on enteroscopic treatment for small bowel DLs. Confirmation of the diagnosis and localization of DL with massive bleeding during BAE is of paramount importance, albeit this process is challenging due to the concurrent drop in blood pressure accompanying active bleeding. Notably, cardiovascular disease and liver cirrhosis have been identified as significant predictors of small bowel angiodysplasia development [18]; however, predictors specific to DL have yet to be revealed, partly due to the insufficient clinical information available from previous studies. In this study, we conducted a thorough review of our database to determine the clinical characteristics and outcomes of patients who underwent DBE as a treatment for small bowel DL bleeding between 2003 and 2022.

## Methods

The present retrospective study was conducted in accordance with the established protocol approved by the Ethics Committee of Nagoya University Hospital, Nagoya, Japan (Approval No. 2015 − 0485). Our clinical approach to the diagnosis of small bowel disease involves the utilization of previously described DBE examinations [19, 20]. The inclusion criteria for DBE in our daily clinical practice focused on OGIB. We generally abstain from using a hood during the DBE procedure, instead opting for a water injection device via the forceps channel. The selection of the peroral and/or transanal routes was based on the location of the suspected lesion, as determined by a comprehensive evaluation encompassing clinical examination, computed tomography (CT) scan, SBCE, and small bowel barium series. In instances in which capsule endoscopy yielded positive findings, the route for DBE was determined by considering the capsule transit time. Conversely, in cases in which capsule endoscopy results were negative or the procedure was not performed, the choice of DBE route was guided by stool color and other imaging modalities.

**Fig. 1:**
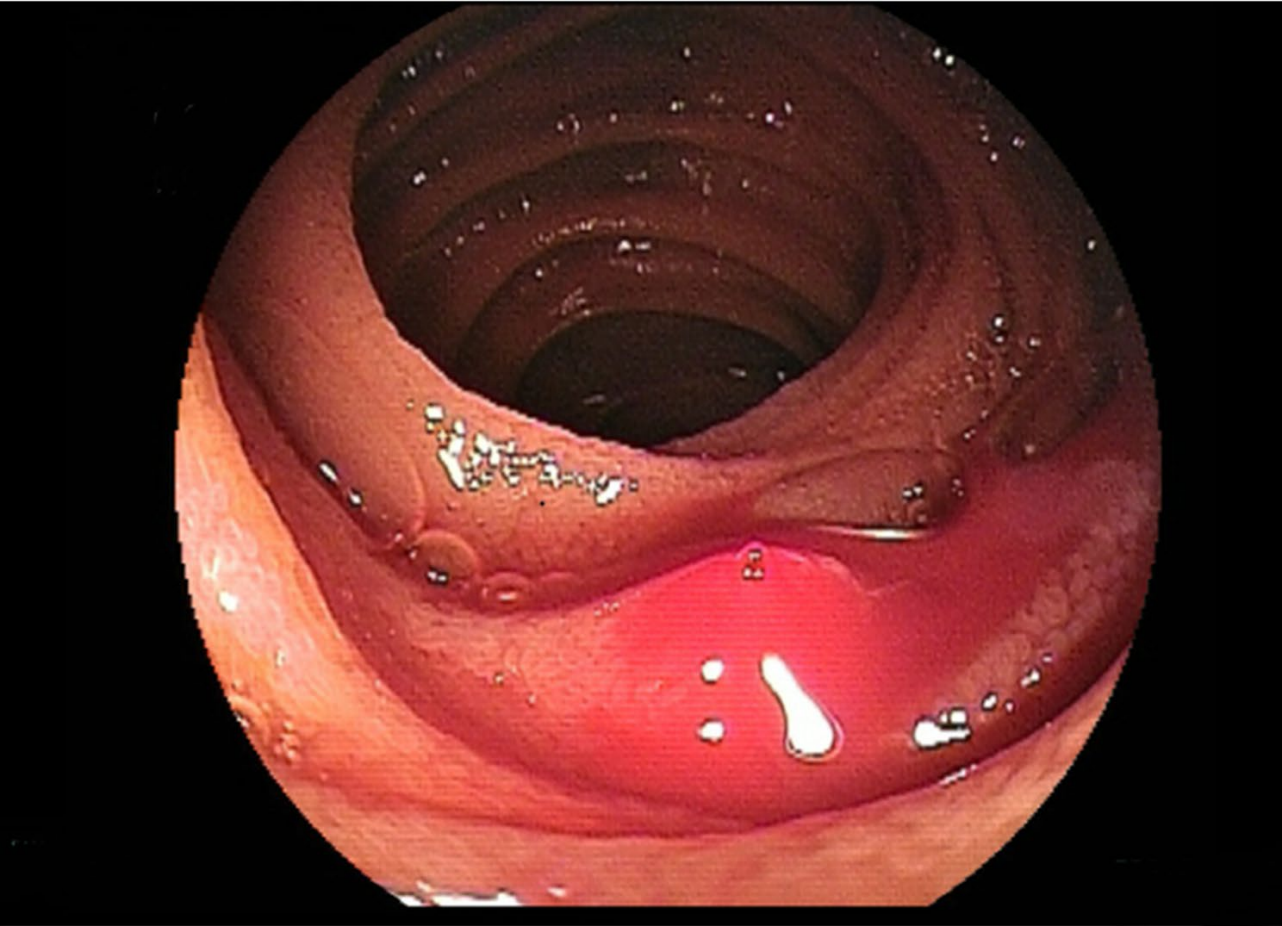
Dieulafoy’s lesion spurting blood from an exposed vessel on the surface that was covered by a clot

**Fig. 2:**
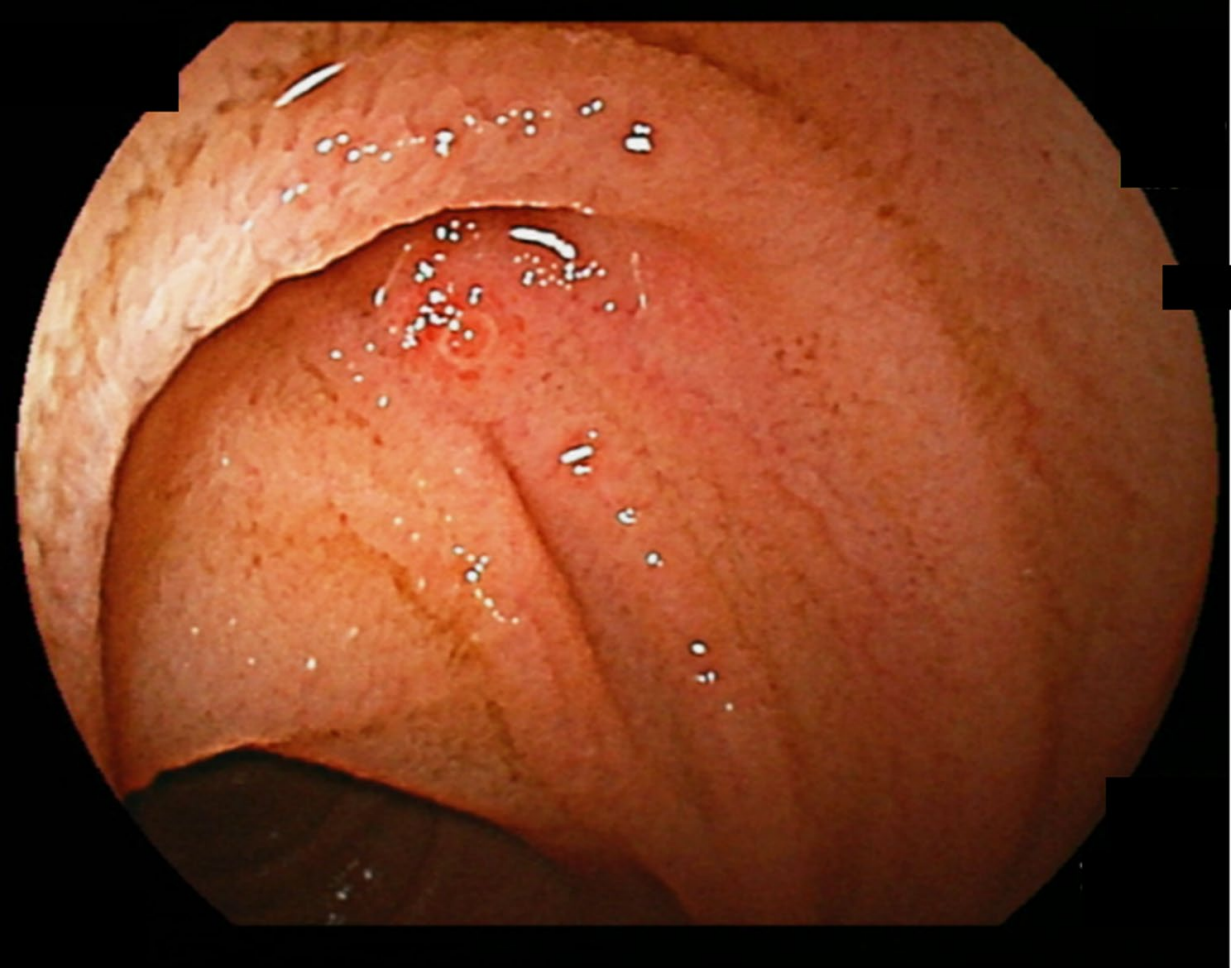
Dieulafoy’s lesion revealing an exposed vessel with red plaque

For the purpose of this study, DL was defined as a small blood vessel exhibiting spurting and active bleeding, or the presence of white or red plaque on the surface without associated mucosal changes, consistent with a Type 2a lesion according to the Yano-Yamamoto classification (see Figs. 1 and 2) [21]. Hematochezia was characterized as overt gastrointestinal bleeding accompanied by bloody stool. In cases in which a DL was not identified during the initial DBE but evidence of hemorrhage was observed, we strategically placed a hemoclip at the most prominent site to facilitate subsequent DBE procedures. Rebleeding was defined as a recurrent bleeding event observed during subsequent DBE, following initial endoscopic treatment.

**Fig. 3:**
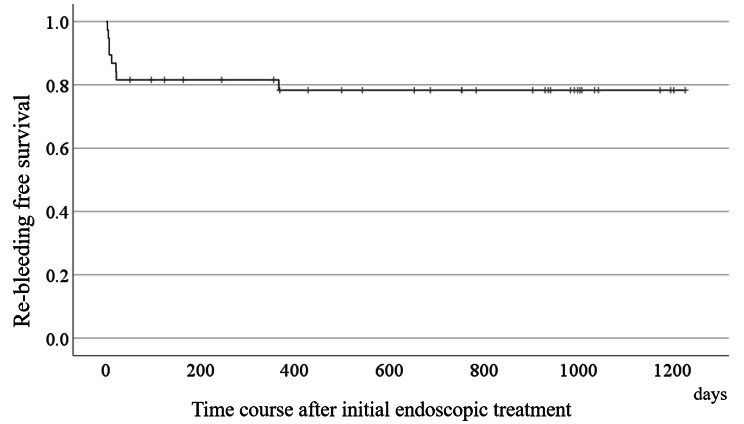
Kaplan-Meier curve for showing the time of rebleeding

A thorough search of the hospital’s database was conducted, identifying a total of 38 patients who were diagnosed with DL and underwent DBE and treatment for suspected small bowel hemorrhage between June 2003 and September 2022. The clinical background, diagnosis, and treatment details of the patients with DL were meticulously examined. We conducted a comparative analysis between the two groups to determine if a diagnosis was established during the initial DBE. Additionally, we conducted a comprehensive multivariate analysis utilizing significant factors to identify the variables that made a substantial contribution to the diagnostic outcome of the first DBE. Furthermore, we performed a two-group comparison between patients who experienced rebleeding following hemostatic treatment and those who did not, followed by a multivariate analysis utilizing the significant factors to identify the variables significantly associated with rebleeding.

### Statistical analysis

Statistical analyses were performed using SPSS software (version 26 for Windows; IBM Corp., Armonk, NY, USA) and StatView software (SAS Inc., Cary, NC, USA). Comparisons between the groups were performed using the Mann-Whitney *U* test or the χ^2^ test, as appropriate. Differences were considered statistically significant at *P* values of < 0.05.

## Results

The characteristics of the patients diagnosed with DL are presented in Table 1. The patients exhibited a variety of underlying medical conditions, with a significant proportion (68%) having a high comorbidity index as indicated by the Ohmiya Index [22]. Prior to their initial visit to our hospital, 16 patients (42%) experienced two or more bleeding incidents. All included patients underwent esophagogastroduodenoscopy (EGD) and total colonoscopy before the first DBE. EGD findings showed mild gastritis in 6 patients, gastric polyps in 5 patients, and reflux esophagitis in 2 patients. Total colonoscopies showed colonic polyps in 9 patients and colonic diverticulum in 3 patients. We determined that any EGD or total colonoscopy did not detect the bleeding origin. While the first DBE confirmed the presence of DL in 23 patients, it failed to detect the condition in the remaining 15 patients, who were subsequently diagnosed through subsequent DBEs (Table 2). The upper jejunum and lower ileum were identified as the most frequent sites affected by small bowel DL (Table 2). The detected DLs exhibited active bleeding (n = 33) and were characterized by exposed vessels with plaque on the surface (n = 5). Out of the 26 CEs performed to address bleeding, 23 showed signs of active bleeding, but the DL itself was not observed in any of the images. The results of CE did not directly correlate with DL detection. In the case of 5 patients who were not initially identified to have DL but exhibited hemorrhage during the first DBE, a hemoclip was placed at the most prominent site to guide subsequent DBEs. Clip placement was employed in the treatment of DLs in 34 patients, while the coagulation method was utilized in 4 patients. We used the hemoclip through the scope, QuickClip 2, manufactured by Olympus Corporation (Tokyo, Japan). Following endoscopic treatment, rebleeding occurred in 8 patients (21%) within a median observation period of 7 days (range: 1-366 days) during a mean observation period of 592 days (Fig. 3). A comparative analysis was conducted between the definitive diagnosis of DL after the first DBE and after two or more DBEs, as shown in Table 3. Lesions situated deep within the duodenum or upper jejunum and the occurrence of two or more episodes of hematochezia were more frequently observed in patients who underwent two or more DBEs. These factors were subjected to multivariate analysis, which revealed that the presence of two or more hematochezia episodes was an independent factor associated with the performance of two or more DBEs for diagnostic purposes (Table 3). The factors associated with rebleeding after endoscopic treatment were analyzed and compared between the rebleeding and non rebleeding groups (Table 4). The initial episode of hematochezia, the number of lesions, and the number of hemoclips used were more frequently observed in the rebleeding group. These factors were subjected to multivariate analysis, which did not identify any independent factors associated with rebleeding. However, it is possible that the number of hemoclips used may have some association (Table 5). No surgical or angiographic embolization cases were observed during the study period.

**Table 1:** Characteristics of patients diagnosed with Dieulafoy’s lesion

	n=38
**Age, years (median range)**	72 (50–92)
**Sex male/female**	19/19
**Underlying disease, n (duplicated)**	
Ischemic heart disease	9
Diabetes mellitus	9
Valvular disease	7
Arrhythmia	7
Chronic kidney disease	7
Liver cirrhosis	6
Heart failure	4
Cerebrovascular disease	2
**Comorbidity Index (Ohmiya index)**	
0 and 1	12
2 and 3	20
4 and more	6
**Long-term use of drugs**	
Anti-thrombus *	22
NSAIDs	4
**Number of events before the first DBE**	
1	22
2	11
≥ 3	5
**Time from onset to DBE**, days (median range)	15 (2–68)
**Lowest Blood hemoglobin level** (g/dl), mean ± SD	5.9 ± 1.6
**Blood transfusion**, units (median range)	10 (0–36)
**Prior capsule endoscopy** (n)	26
**Number of DBEs until confirmed diagnosis**	
1	23
2	13
3	1
5	1

DBE, double-balloon endoscopy

* Antithrombotic drugs included warfarin in 11 patients, low-dose aspirin in 6 patients, Clopidogrel in 2 patients, Ticlopidine in 2 patients, and Prasugrel Hydrochloride in 1 patient. They were duplicated

**Table 2:** Characteristics of the lesions and treatment

	n=38
Distribution (duplicated)*	
Deep duodenum	3
Upper jejunum	**18**
Middle jejunum	**8**
Lower jejunum	**2**
Middle ileum	**2**
Lower ileum	**6**
**Bleeding status**	
Active bleeding	**33**
Small blood plaque on the surface	**5**
**Single lesion**	**32**
Angiodysplasia complicated**	**13**
**Endoscopic Treatment**	
Hemoclips placement	**34**
Coagulation	**4**
**Number of used hemoclips (mean±SD)**	**2.7 ± 1.6**
**Rebleeding during follow-up, n**	**8**

SD: Standard Deviation

*The distribution of lesions was determined by two endoscopists who referred to following articles [38, 39]

** Angiodysplasia was found during the observation range during double-balloon endoscopy

**Table 3:** Multivariate analyses of the factors to confirm diagnosis of Dieulafoy’s lesion after the first double-balloon endoscopy

Patient characteristics	Definite diagnosis		*P-*value		multivariate analyses		
	first DBE	≥ 2 DBEs			*P-*value	OR	95% CI
N	23	15					
**Gender**			0.091		0.23	0.4	0.09‒1.78
male	9	10					
female	14	5					
**Age, mean±SD**	70±10	72±10	0.445				
**Comorbidity (Ohmiya Index)**	2.2±1.5	2.3±2.1	0.725				
**Maintenance medications**							
**Antithrombotic agents**			0.901				
yes	14	8					
no	9	7					
**NSAIDs**			0.64				
yes	3	1					
no	20	14					
**Prior capsule endoscopy**			0.958				
performed	16	10					
not performed	7	5					
**Lowest hemoglobin level**	5.9±1.3	6.1±2.1	0.686				
(mean±SD, g/dl )							
**Blood transfusion**	11.7±9.7	11.0±6.2	0.651				
(mean±SD, units )							
**Days from Onset to endoscopy**	23±19	24±16	0.384				
(mean±SD, days)							
**First episode of hematochezia**			0.02		0.03	5.37	1.09‒26.33
yes	17	5					
no	6	10					
**Lesion located in the duodenum or**			0.02		0.82	1.2	0.23–6.07
**upper jejunum**							
yes	9	12					
no	14	3					
**Number of lesions**			0.663				
single	20	12					
two or more	3	3					
**Angiodysplasia complicated**			0.338				
yes	6	7					
no	17	8					

OR, odds ratio; CI, confidence interval

**Table 4:** Comparison of the rebleeding and non rebleeding groups

Patient characteristic	Rebleeding		*P* value
	Occurred	Not occurred	
N	8	30	
**Sex**			0.99
Male	4	15	
Female	4	15	
**Age, mean ± SD**	73 ± 10	70 ± 10	0.542
**Comorbidity (Ohmiya Index)**	2.6 ± 3.0	2.2 ± 1.3	0.673
**Drugs use**			
**antithrombotic**			0.563
Yes	8	25	
No	0	5	
**NSAIDs**			0.99
Yes	1	3	
No	7	27	
**Prior capsule endoscopy**			0.99
Performed	6	20	
Not performed	2	10	
**Lowest hemoglobin level**	6.2 ± 3.0	5.9 ± 1.1	0.36
(mean ± SD, g/dl)			
**Blood transfusion, units**	12.5 ± 6.3	11.2 ± 9.0	0.226
(mean ± SD)			
**Days from Onset to endoscopy**	22 ± 17	23 ± 18	0.969
(mean ± SD)			
**First episode of hematochezia**			0.049
Yes	2	20	
No	6	10	
**Lesion located in the duodenum or**			0.708
**upper jejunum**			
Yes	5	16	
No	3	14	
**Number of lesions**			0.011
1	4	28	
≥ 2	4	2	
**Angiodysplasia complicated**			0.999
Yes	3	10	
No	5	20	
**Diagnosed by the first DBE**			0.686
Yes	4	19	
No	4	11	
Number of used hemoclips	3.5 ± 0.7	2.5 ± 1.7	0.043
**Small blood clot on the surface**			0.563
Yes	0	5	
No	8	25	

NSAID, nonsteroidal anti-inflammatory drugs; DBE, double-balloon endoscopy; SD: standard deviation

**Table 5:** Multivariate analyses of factors affecting rebleeding after endoscopic treatment

	*P* value	OR	95% CI
Multiple lesions	0.16	0.18	0.01‒1.98
Number of used hemoclips	0.06	7.39	0.85‒63.95
s or more episodes of hematochezia	0.26	3.11	0.42‒23.10

OR, odds ratio; CI, confidence interval

## Discussion

Several case series have indicated that DL is more commonly underdiagnosed than considered a rare condition [23–26]. The primary objective of this study was to elucidate the clinical characteristics of small bowel DLs, which pose considerable challenges in terms of diagnosis. Our analysis revealed that many patients with DL had underlying diseases, and the comorbidity index of the patients was relatively high, consistent with previous reports [3, 22] (Table 1). Intestinal vessel diseases affecting the small bowel, such as angiodysplasia and ischemic enteritis, often coexist with severe underlying conditions and are associated with peripheral vein disorders [3, 19]. Although DL is an arterial disease, it is also influenced by underlying medical conditions. Although there is limited evidence for indicating the pathology of OGIB and the peculiarities of the bleeding source within the GI tract, DLs may offer valuable insights into the pathophysiology of OGIB. In our study, DLs disappeared in 15 out of 38 patients (39.4%) after the initial DBE procedure, despite thorough searching for the lesion site. The median time from the onset of bleeding to DBE was 15 days in this study. In clinical practice, a similar duration may elapse before DL diagnosis through DBE. The independent factor associated with failed DL diagnosis during DBE was the occurrence of two or more episodes of hematochezia (Table 3). For such patients, careful consideration should be given to the timing of examinations and the use of other supportive diagnostic modalities. Emergency SBCE, a noninvasive procedure, can be performed for ongoing OGIB and can yield high diagnostic rates [27]. However, this procedure is not feasible for patients with a full stomach. In such cases, the administration of erythromycin can aid in visualization and capsule transit [28]. Enhanced CT scans can provide information about the localization of the bleeding site, enabling prompt scheduling of antegrade or retrograde BAE.

Regarding the treatment of DLs, rebleeding after treatment occurred in 8 out of 38 patients (21%). Although the observation period was not lengthy, this rate was higher than the rebleeding rate reported in other studies on vascular lesions [3]. Notably, rebleeding events were observed several days after endoscopic treatment during the admission period (Fig. 3). Therapeutic options for rebleeding include repeat endoscopic hemostasis, angiographic embolization, or surgical wedge resection of the lesions. Typically, endoscopic and angiographic interventions are initially attempted to control bleeding from DLs, with surgical approaches reserved for cases where other methods have failed [29]. Angiographic embolization can stabilize vital signs and effectively halt bleeding. Surgical resection was performed in only 5% of patients [10].

We analyzed the factors influencing rebleeding after endoscopic treatment; however, no independent factor was identified (Table 5). The number of hemoclips used may have some influence. If the initial clip placement is unsuccessful, additional clips may be employed. In contrast, endoscopic management of DLs has been shown to be highly effective as both a diagnostic and therapeutic approach in other studies [30]. Lipka et al. reported no adverse events among eight patients and demonstrated that none of the patients required surgery following treatment of small bowel DLs with single-balloon enteroscopy [29]. After the failure of initial endoscopic treatment, the utility of optional devices in endoscopy, such as endoscopic band ligation and the over-the-scope clip system, has been reported [31, 32]. Malik et al. presented different results regarding DL management compared to this study [8]. In their study, male patients were predominant, with a median age of 55 years. The main symptom was melena, and overt GI bleeding was observed during the initial diagnosis. Endoscopic treatment was successful in 64% of patients, while 34% of endoscopists opted for a combination therapy involving two or more endoscopic modalities. The rebleeding rate was 13.4%, and the overall mortality rate was 4.4%. The authors suggested that jejunal DL represents a challenging etiology, and the time interval between initial presentation and diagnostic modality is crucial for early diagnosis [16].

Our study specifically focused on DLs and had a selection bias toward small bowel vascular lesions. One study identified age, aortic stenosis, chronic renal diseases, chronic obstructive pulmonary disease, venous thromboembolism, cardiovascular disease, and liver cirrhosis as risk factors for the development of small bowel angiodysplasia [33]. Another study reported that cardiovascular disease and liver cirrhosis were present in 40% and 23% of cases, respectively, among patients with small bowel angiodysplasia. The rebleeding rates for small bowel angiodysplasia in Yano-Yamamoto classification types 1a and 1b were 6% and 17%, respectively [18]. DL shares similarities with small bowel angiodysplasia in terms of underlying baseline diseases, and the rebleeding rate may be higher than that of small bowel angiodysplasia. Additionally, we collected data on DLs defined as Yano-Yamamoto classification type 2a, which are characterized as punctuate vascular lesions smaller than 1 mm, based on the DBE database. Distinguishing DL from vascular or arteriovenous malformations can sometimes be challenging. In such cases, the Yano-Yamamoto classification system can aid in differentiation in clinical practice. Non-small bowel lesions are occasionally identified as the source of OGIB [34, 35]. In our study, EGD and total colonoscopy were performed in all patients, but they did not reveal the origin of the digestive bleeding. Furthermore, no other source of OGIB was found during the observation period after DBE.

This study had several limitations. First, it was a retrospective study with a small number of patients. The definition of hematochezia relied on clinical information provided by bleeding patients and clinicians, rather than objective criteria. The decision to perform DBE was based on the physician’s discretion. Some patients were not followed up after treatment due to a lack of additional medical information in their charts or other reasons. The number of patients in whom DL diagnosis was missed during DBE is unknown. We identified DLs in the deep duodenum in three patients (7.8%), which were not detected during previous upper GI endoscopy. However, with advancements in endoscopic devices and techniques, reaching the horizontal section of the duodenum with the endoscope has become feasible in recent years. In such cases, these lesions could be identified much earlier using upper GI endoscopy [36, 37]. The identification of predictors for DL was not the focus of this study, as our aim was to elucidate the characteristics of DL through a case series.

In conclusion, for patients experiencing two or more episodes of hematochezia, prompt scheduling of BAE, facilitated by optional devices, is crucial, especially in the presence of underlying diseases, after standard endoscopic evaluation with esophagogastroduodenoscopy and colonoscopy is unrevealing.

## Data Availability

The datasets used and/or analyzed during the current study are available from the corresponding author on reasonable request.
